# Protective effects of methanol extract of *Plukenetia conophora* seeds and 4H-Pyran-4-One 2,3-Dihydro-3,5-Dihydroxy-6-Methyl on the reproductive function of male Wistar rats treated with cadmium chloride

**DOI:** 10.5935/1518-0557.20180048

**Published:** 2018

**Authors:** Olugbemi Tope Olaniyan, Olufadekemi Tolulope Kunle-Alabi, Yinusa Raji

**Affiliations:** 1 Department of Physiology, Bingham University Karu, Nasarawa State, Nigeria; 2 Department of Physiology, University of Ibadan, Ibadan, Oyo State, Nigeria

**Keywords:** *Plukenetia conophora*, cadmium chloride, 4H-Pyran-4-One 2,3-Dihydro-3,5-Dihydroxy-6-Methyl, male reproduction, Wistar rats

## Abstract

**Objectives:**

Male infertility caused by exposure to heavy metals is a current global issue. Exposure to cadmium chloride (CdCl_2_) negatively affects the male reproductive system. Many infertile people, especially in developing countries, resort to folkloric treatment. *Plukenetia conophora* is used in Nigerian folk medicine to promote fertility. This study investigated the effects of *Plukenetia conophora* (PC) and 4H-Pyran-4-One 2,3-Dihydro-3,5-Dihydroxy-6-Methyl (DDMP) on Wistar rats with cadmium chloride-induced testicular damage.

**Methods:**

Forty-two male Wistar rats (150-190g) were divided into seven groups (n=6) and treated daily for 54 days as follows: Controls (normal saline); CdCl_2_ (2mg/kg single IP dose); CdCl_2_ + 200 mg/kg vitamin E; CdCl_2_ + 100 or 200 mg/kg PC; and CdCl_2_ + 25 or 50 mg/kg DDMP. The rats were sacrificed 55 days after the start of the study; Samples were collected for analysis. Biochemical parameters malondialdehyde, nitric oxide, antioxidant enzymes, and proton pumps were measured by spectrophotometry. Reproductive hormones were measured using ELISA. Data were analysed using ANOVA and differences in mean values were considered significant at *p*<0.05.

**Results:**

Significant increases in sperm count, motility, and viability were observed in the groups given CdCl_2_+Vitamin E, CdCl_2_+PC or CdCl_2_+DDMP as compared with the CdCl_2_ group. Malondialdehyde and nitric oxide levels in the groups treated with CdCl_2_+PC or CdCl_2_+DDMP decreased significantly when compared with the group given CdCl_2_. Significant increases were observed in antioxidant enzymes, proton pump, and testosterone in the groups treated with CdCl_2_+PC or CdCl_2_+DDMP, respectively.

**Conclusion:**

*Plukenetia conophora* alleviated male reproductive toxicity induced by cadmium chloride in Wistar rats. 4H-Pyran-4-One 2,3-Dihydro-3,5-Dihydroxy-6-Methyl present in *Plukenetia conophora* may be responsible for the ameliorative effects.

## INTRODUCTION

Infertility is a global issue that affects 13-15% of couples worldwide (Agarwal *et al.,* 2004). The World Health Organization (WHO) defines infertility as the inability of a sexually active couple off contraceptives to achieve spontaneous pregnancy in one year of unprotected sexual intercourse (WHO, 2010). However, male factor infertility accounts for up to half of all cases of infertility and affects one in 20 men of the general population (McLachlan & de Krester, 2001). Over the last decades, a significant decrease in human fertility has been observed and there is no doubt that modern lifestyle affects the fertility level of every male (Benoff *et al.,* 2000). Several factors might be responsible for this, including exposure to heavy metals (Benoff *et al*., 2008).

The toxic effect of drugs and environmental chemicals on the human reproductive system has become a major health concern. Cadmium (Cd) is a heavy metal toxicant, present widely in our environment (Akinloye *et al.,* 2006). The production of nickel-cadmium batteries is a very significant source of Cd. It is well known that cadmium causes adverse effects on the male reproductive function of experimental animals. It produces a wide range of biochemical and physiological dysfunctions in humans and laboratory animals (Santos *et al.,* 2004). The release of cadmium into the environment increased significantly in most industrialized countries during the second half of the last decade (Horiguchi *et al.,* 1996). Exposure to cadmium may negatively affect the male reproductive system via degenerative changes in the testes, epididymis, and seminal vesicle. Cadmium induces testicular lipid peroxidation by generating free radicals, thereby impairing the intracellular defence system (Agarwal *et al.,* 2004). Studies showed that sperm damage mediated by reactive oxygen species (ROS) is a significant contributing factor in 30-80% of all infertility cases (Agarwal *et al.,* 2004). To combat oxidative stress, the body has evolved several antioxidant systems. However, these systems may be overwhelmed by excessive generation of ROS. Hence, antioxidant supplements are often needed.

Medicinal plants often exhibit a wide range of biological and pharmacological activities that translate into anti-inflammatory, anti-bacterial, anti-fungal, and antioxidant properties (Okwu & Ekeke, 2003). A number of medicinal plants are of common use in African traditional medicine. One of them is *Plukenetia conophora* (commonly called African walnut). Extracts from its roots, bark, seeds, and fruit are used in the preparation of syrups and infusions in traditional medicine to treat ailments such as coughs, liver cirrhosis, and hepatitis (Iwu, 1986).

*Plukenetia conophora*leaves have good free radical scavenging activity (Amaeze *et al.,* 2011). *Plukenetia conophora* is a rich source of polyphenols, which are antioxidants in nature and have recently attracted considerable attention for preventing oxidative stress-related diseases such as cancer, cardiovascular disease, degenerative disease, and infertility. Antioxidant properties, ROS scavenging, and cell function modulation of flavonoids account for the large part of pharmacological activity. The presence of a wide range of phytochemical constituents in the seed extract of *Plukenetia conophora* indicates that the plant might be used in a multitude of beneficiary ways than already studied. The present study was carried out to evaluate the effect of methanol extract of *Plukenetia conophora* seeds and its flavonoid fraction (4H-Pyran-4-One 2,3-Dihydro-3,5-Dihydroxy-6-Methyl (DDMP) against CdCl_2_-induced testicular damage in Wistar rats.

## MATERIALS AND METHODS

*Plukenetia conophora* fruits were harvested from a farm at Okeho, Oyo State, Nigeria. They were identified at the Forestry Research Institute of Nigeria (FRIN), Ibadan, Oyo State, Nigeria, against specimen No. FHI 109997. The fruits were de-shelled and the collected seeds were air-dried. The air-dried seeds were extracted by cold maceration in methanol, and evaporated to dryness on a rotary evaporator (rotavap R-200) at reduced temperature.

### Phytochemical Screening

Methanol extract of *Plukenetia conophora* seeds and the powdered seeds were subjected to preliminary phytochemical screening for the detection of various plant constituents using methods described in the literature (Sofowora, 1982; Trease & Evans, 1989). The screening procedures were carried out at the Department of Pharmacology, Faculty of Pharmacy, University of Jos, Nigeria.

### Isolation and Characterisation

Column chromatography was carried out on silica gel (70-230 and 240-300 mesh size, Merck, Germany), Merck alumina (70-230 mesh). Thin layer chromatography was carried out on pre-coated silica gel 60 F254 aluminium foil (Merck, Germany) to establish the purity of the isolates. Spots on TLC were examined with a UV lamp operating at a wavelength of 365 nm for fluorescence and at 254 nm for fluorescence quenching spots. The brown tail seen when using the UV light at 254 nm indicated the position of flavonoids.

Characterisation was done using three spectral analyses. The isolate obtained as described above was characterised using Fourier Transform Infrared (FTIR) spectroscopy, ultraviolet spectrophotometry, and Gas Chromatography-Mass Spectrometry (GC-MS).

### Identification of components

Interpretation of mass spectrum GC-MS was carried out by comparing the database peaks from the National Institute Standard and Technology (NIST) library with the values reported in the literature (Schauer *et al.,* 2005; Tsivou *et al.,* 2006). The spectrum of unknown compounds was compared with the spectrum of known compounds stored in the NIST library.

### Toxicity evaluation of methanol extract of *Plukenetia conophora* seeds

The acute toxicity tests of *Plukenetia conophora* seed extract were carried out according to the Organization of Economic Co-operation and Development (OECD) Test Guidelines (OECD 423- Limit test procedure) (OECD, 2001).

### Brine shrimp lethality assay for LC _50_

Brine shrimp eggs (*Artemia salina*) were obtained from the Department of Pharmacognosy, University of Ibadan. They were hatched in natural seawater obtained from the bar beach, Ikoyi, Lagos, Nigeria and incubated for 48 hours in 3.8 g/l seawater. After hatching, the nauplii were collected and treated with selected concentrations (five dilutions, 0.01-1000 mg/ml) of plant extracts and cyclophosphamide as the standard drug. A plastic chamber with two unequal compartments separated with a divider with several holes was used for hatching. The eggs were sprinkled into the larger non-illuminated compartment, while the smaller compartment was illuminated. After 48 hours of incubation at room temperature (25-29ºC), the nauplii (larvae) were collected using a Pasteur pipette from the lighted side, whereas their shells were left in the other side. The procedure for BSLT was modified from the assay described by Solis *et al.* (1993). Ten milligrams of the extract were made up to 1 mg/ml in artificial seawater. Serial dilutions were made in the wells of 96-well microplates in triplicate in 120µl seawater. Control wells with distilled water were included in each experiment. A suspension of nauplii containing 10-15 organisms (100µl) was added to each well. The plates were covered and incubated at room temperature (25-29ºC) for 24 hours. The plates were examined on a binocular stereomicroscope and the dead (non-motile) nauplii in each well were counted. One hundred microliters of methanol were added to each well to immobilise the nauplii, and after 15 minutes the total number of brine shrimps in each well was counted. Analysis of the data was performed using the Graph pad prism computer program to determine the lethal concentration to half of the test organisms (LC_50_).

### Experimental Animals and Handling

Forty-two adult male Wistar rats weighing 150-190g were obtained from the Central Animal House, College of Medicine, University of Ibadan. They were acclimatized for two weeks before the start of the experiment. Hard wood beddings (saw dust) were used. They received food and water *ad libitum* throughout the period of the experiment.

### Experimental protocol

The animals were randomly divided into seven groups of six rats each as follows:

**Group I -** Control animals administered normal saline orally.
**Group II -** Animals given a single IP dose of 2 mg/kg of BW of cadmium chloride.
**Group III -** Animals pre-treated with a single IP dose of 2 mg/kg of BW of cadmium chloride and then given 200 mg/kg of BW of vitamin E orally.
**Group IV** and **V -** Animals pre-treated with a single IP dose of 2 mg/kg of BW of cadmium chloride and then given 100 or 200 mg/kg of BW of PC orally, respectively.
**Group VI** and **VII -** Animals pre-treated with a single IP dose of 2 mg/kg of BW of cadmium chloride and then given 25 or 50 mg/kg of BW of DDMP orally, respectively.

### Sample Collection

Blood samples were collected via the retro-orbital venous sinus (Van Herck *et al.,* 1992) and serum was obtained for the determination of male sex hormones (Follicle Stimulating Hormone, Luteinizing Hormone, and Testosterone). The rats were then sacrificed by cervical dislocation. The testes, epididymis, and seminal vesicles were collected, cleared of adherent tissue and weighed using a sensitive weighing scale.

### Sperm analysis

Epididymal contents were collected by cutting the cauda epididymis and squeezing it gently on a clean slide. Sperm progressive motility and cell count were determined according to the method described by Yokoi *et al.* (2003). Briefly, cauda epididymis specimens were minced with anatomical scissors in 2 ml of Earles’ buffer and placed in a rocker for 10 min at 37ºC. After dilution, the number of homogenization-resistant spermatozoa was counted in a haemocytometer and about 25 fields of view were examined under a light microscope at 40× magnification.

### Lipid peroxidation assay

Malondialdehyde (MDA) levels were estimated by the method described by Kartha & Krishnamurthy (1978). MDA was measured as an indicator of lipid peroxidation and ROS by extension. Serum samples were placed in a micro-centrifuge tube and incubated with thiobarbituric acid (TBA). Following incubation, the samples were centrifuged (2000 rpm, 10 minutes) and the absorbance of the pink clear supernatant was measured at 532 nm in duplicate samples. Malondialdehyde bis-(dimethyl acetal) was used as the external standard. Thiobarbituric acid reactive substances were expressed in terms of nanomoles of MDA/gram of wet tissue. Lipid peroxidation was determined by measuring the thiobarbituric acid reactive substances (TBARS) produced during lipid peroxidation (Varshney & Kale, 1990).

### Determination of tissue superoxide dismutase activity

Superoxide dismutase (SOD) was estimated by the technique explained by Fridovich (Beauchamp & Fridovich, 1971). Activity was expressed as unit/mg of protein. The level of SOD activity was determined by the method described by Misra & Fridovich (1972). The ability of SOD to inhibit the autoxidation of epinephrine at pH 10.2 makes this reaction the basis for a simple assay for superoxide dismutase. The superoxide (O_2_^•−^) radical generated by the xanthine oxidase reaction causes the oxidation of epinephrine to adrenochrome and the yield of adrenochrome produced per introduced O_2_^•−^ increases with pH (Valerino & McCormack, 1971) and with the concentrations of epinephrine. These results led to the proposal that the autoxidation of epinephrine proceeds by at least two distinct pathways, only one of which is a free radical chain reaction involving the superoxide (O_2_^•−^) radical and hence subject to inhibition by SOD.

### Determination of tissue catalase activity

Catalase activity was determined according to the method described by Sinha (1972). This method is based on the fact that dichromate in acetic acid is reduced to chromic acetate when heated in the presence of H_2_O_2_, with the formation of perchromic acid as an unstable intermediate. The chromic acetate then produced is measured by colorimetric analysis at 570-610 nm. Since dichromate has no absorbency in this region, the presence of the compound in the assay mixture does not interfere at all with the colorimetric determination of chromic acetate. The catalase preparation is allowed to split H_2_O_2_ for different periods of time. The reaction is stopped at a particular time with the addition of a dichromate/acetic acid mixture and the remaining H_2_O_2_ is determined by measuring chromic acetate by colorimetric analysis after heating the reaction mixture.

### Estimation of glutathione peroxidase

Glutathione peroxidase was estimated based on the procedure described by Rotruck *et al.* (1973). The whole reaction mixture was incubated at 37ºC for 3 minutes; then 0.5 ml of trichloroacetic acid was added and the mixture was centrifuged at 3000 rpm for 5 minutes. To 1 ml of each of the supernatants, 2 ml of K_2_HPO_4_ and 1 ml of DTNB were added and absorbance was read at 412 nm against a blank. Glutathione peroxidase activity was observed by plotting the standard curve and the concentration of the remaining GSH was extrapolated from the curve.

GSH consumed = 245.34 - GSH remaining

Glutathione peroxidase activity = H_2_0_2_ consumed/mg of Protein

### Determination of tissue glutathione-S-transferase activity

Glutathione-S-transferase (GST) activity was determined according the method described by Habig *et al.* (1974). The method is based on the principle that glutathione-S-transferase demonstrates a relatively high level of activity in the presence of 1-chloro-2,4-dinitrobenzene (CDNB), the substrate used in the assay to measure GST activity. When CDNB is conjugated with reduced glutathione, the absorption maximum shifts to a longer wavelength. The absorption increase at the new wavelength of 340 nm provides a direct measurement of the enzymatic reaction.

### Total protein (Biuret reagent)

Protein content of the tissue samples was determined using the method described by Lowry *et al.* (1951).

### Assay procedure

In alkaline medium, copper reacts with the peptide bonds of proteins to form a characteristic pink to purple biuret complex. Potassium sodium tartrate prevents the precipitation of copper hydroxide, and potassium iodide prevents the auto reduction of copper.

Protein+Cu2AlkalinepH−−−−−−−−>Cu−Proteincomplex

The colour intensity is directly proportional to the protein concentration. It is measured based on the increase in absorbance at 546 nm using spectrophotometry (F93 Drawell fluorospectrophotometer).

### Determination of tissue nitric oxide (NO) content

### Total nitrite

One millilitre of 1 mM nitrite was dissolved in 9 ml of distilled water to yield a 100-NM nitrite standard. Then 1% sulfanilic acid was added to 50 µl of standard, and the mixture was incubated for 5-10 minutes. Afterwards, 1% N-(1-Naphthyl)ethylenediamine dihydrochloride (NED) was added and incubated for 5-10 minutes to allow for colour development. The resultant mixture was read on a spectrophotometer (F93 Drawell fluorospectrophotometer) at 540 nm. Standards were replaced with samples (50 µl).

### Estimation of serum testosterone, luteinizing hormone, and follicle stimulating hormone

The serum samples obtained were analysed to determine the concentrations of testosterone, luteinizing hormone, and follicle stimulating hormone. The analysis was carried out via tube-based enzyme immunoassay (EIA) method. The protocol used in hormone testing followed the method described by the kit manufacturers (Immunometrics Limited UK) and met the WHO research programme standards for reproductive studies.

### Determination of testicular and epididymal proton pump (ATPase) activity

Na^+^/K^+^-ATPase, calcium ATPase, and magnesium ATPase activities were analysed based on a modification of the method published by Evans (1969). Spectrophotometry was used to measure the levels of inorganic phosphate in the testicular and epididymal tissue homogenate as per the method described by Bonting (1970).

### Statistical Analysis

Data were presented as mean values ± SEM. ANOVA was carried out on the Statistical Package for the Social Sciences (SPSS package version 18) and the occurrence of significant differences between the results was verified. Differences were deemed significant at *p*<0.05.

### RESULTS

### Phytochemical screening of the methanol extract of *Plukenetia conophora* seeds

The results for the phytochemical screening of the methanol extract of *Plukenetia conophora* seeds against the preparation with powdered seeds revealed that flavonoids, carbohydrates, steroids, and cardiac glycosides were moderately to highly present in the methanol extract of *Plukenetia conophora* seeds as compared with the powdered extract, whereas alkaloids were highly present in the powdered extract as compared with the methanol extract. The screening tests also showed that saponins, tannins, resins, and anthraquinones were absent in both preparations (Table 1).

**Table 1:** Phytochemical Screening showed the presence of the following active constituents.

Phytochemicals	MEOH Extract	Powdered extract	Description
Alkaloids	++	+++	Red ppt –Draggendroffs Milky – Mayer’s reagent
Saponins	-	-	
Tannins	-	-	
Flavonoids	++	+	yellow ppt
Carbohydrates	+++	+	violet
Cardiac glycosides	+++	++	brick red ppt
Steroids	+++	++	brick red ppt
Anthraquinones	-	-	
Resins	-	-	

- negative

+ present

++ moderately present

+++ highly present

### Spectroscopic flavonoid fraction analysis

**Figure 1:**
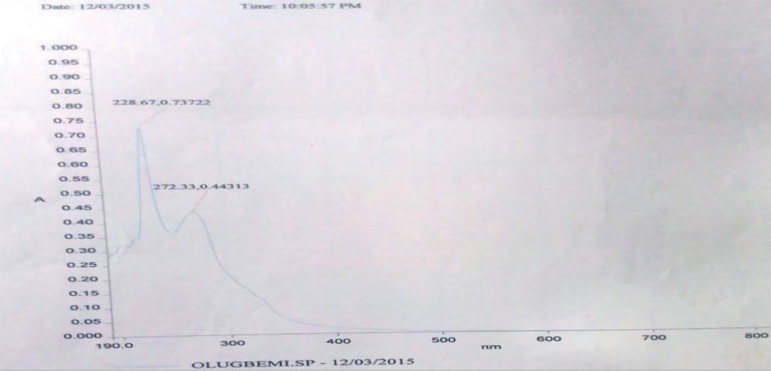
Ultraviolet (UV) spectrophotometry analysis of the *flavonoid fraction*

Ultraviolent-visible spectroscopy showed two wavelengths of maximum absorbance at 228.67 and 272.33 nm consistent with the presence of isolated chromophores. The chromophores were α and β- unsaturated ketone groups on the *flavonoid fraction* (Figure 1).

### Fourier Transform Infrared (FTIR) analysis of the flavonoid fraction and Gas Chromatography-Mass (GC-MS) Spectrometry analysis

**Figure 2:**
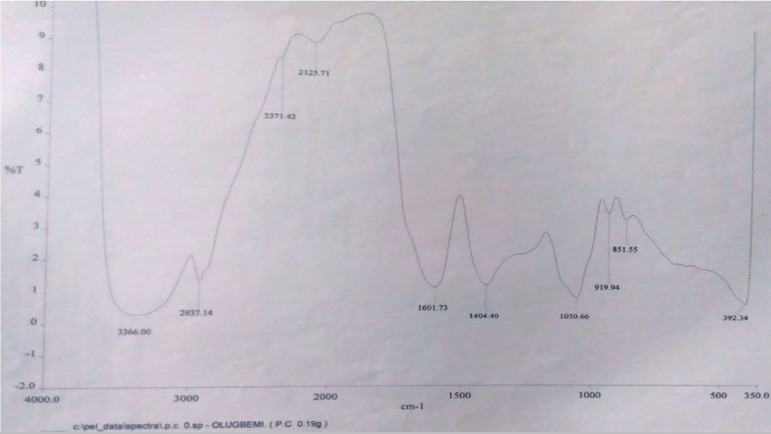
Fourier Transform Infrared (FTIR) analysis of the *flavonoid fraction*

**Figure 3:**
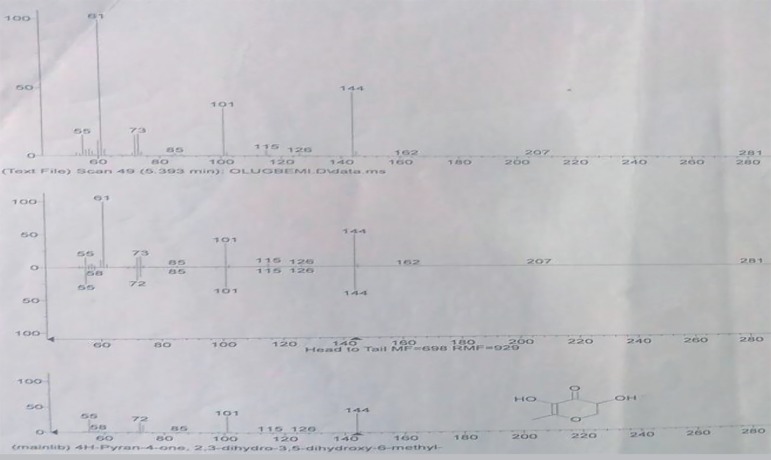
Structure of the major flavonoid fraction 4H-Pyran-4-One 2,3-Dihydro-3,5-Dihydroxy-6-Methyl in GC-MS analysis

Fourier Transform Infrared analysis of the flavonoid fraction confirms the presence of principal functional groups indicated by the vibrations of 3366 cm^-1^ characteristically seen in the presence of the O-H stretch of free hydroxyl groups; 2937.14 cm^-1^, indicative of C-H stretch of alkanes; and 1601.73 cm ^-1^, indicative of α and β unsaturated ketone groups (Figure 2). These key vibrations were consistent with the functional groups found on 4H-Pyran-4-One 2,3-Dihydro-3,5-Dihydroxy-6-Methyl as revealed by GC-MS analysis (Figure 3).

### Acute oral toxicity test of methanol extracts of *Plukenetia conophora* seeds

No deaths were recorded from the administration of methanol extracts of *Plukenetia conophora* seeds in dosages of up to 3200 mg/kg of body weight (Table 2). Higher dosages were not tested to estimate the LD_50_ (lethal dose) value. According to the OECD guidelines for acute toxicity, LD_50_ dosages of 2000 mg/kg of body weight and greater are deemed safe for rats.

**Table 2:** Acute oral toxicity of methanol extracts of Plukenetia conophora seed.

Doses (mg/kg)	Mortality rate (%)
100	0
200	0
400	0
800	0
1600	0
3200	0

n=3

Total number of deaths = 0

### Lethal concentration (LC_50_)of 4H-Pyran-4-One 2,3-Dihydro-3,5-Dihydroxy-6-Methyl

No deaths were recorded from the administration of 4H-Pyran-4-One 2,3-Dihydro-3,5-Dihydroxy-6-Methyl in dosages of up to 1000 µg/ml (Table 3). Higher dosages were not tested to estimate the LC_50_ (Lethal Concentration) value. According to the guidelines for toxicity testing using brine shrimp lethality tests as described by Solis *et al.* (1993), LC_50_ dosages of 1000µg/ml and greater are deemed safe for rats.

**Table 3:** LC_50_ of 4H-Pyran-4-One 2,3-Dihydro-3,5-Dihydroxy-6-Methyl.

Conc. (µg/ml)	% Survival			% Death		
1	2	3	1	2	3
1000	100	100	100	0	0	0
500	100	100	100	0	0	0
100	100	100	100	0	0	0
10	100	100	100	0	0	0
1	100	100	100	0	0	0

LC _50_>1000 µg/ml

### Effects of oral administration of *Plukenetia conophora* and 4H-Pyran-4-One 2,3-Dihydro-3,5-Dihydroxy-6-Methyl on body weight

The results of this study showed that the group treated with cadmium chloride had a significant reduction (*p*<0.05) in body weight gain as compared with controls, a finding also reported in another study (de Souza Predes *et al.,* 2010). The group pre-treated with cadmium chloride and administered oral *Plukenetia conophora* or 4H-Pyran-4-One 2,3-Dihydro-3,5-Dihydroxy-6-Methyl showed improvement in body weight gain as compared with the group given cadmium chloride only (Table 4).

**Table 4:** Effects of oral administration of Plukenetia conophora seed and 4H-Pyran-4-One 2,3-Dihydro-3,5-Dihydroxy-6-Methylon the body weight of male Wistar rats treated with a single IP dose of 2 mg/kg of cadmium chloride.

Group	Body weight (g)		
Initial	Final	Gain in Body weight
Control	75.70±1.75	182.50±6.45	57.74±1.26
Cadmium chloride	109.50±4.06	155.70±9.67	23.89±3.34^+^
Cd+Vit E	141.30±10.13	191.30±23.71	25.76±8.64
Cd+100 mg/kg PC	107.50±4.03	155.80±8.01	26.53±2.01
Cd+200mg/kg PC	104.20±1.08	159.80±6.23	34.43±2.60
Cd+25 mg/kg DDMP	101.30±3.17	188.70±6.70	45.28±3.26
Cd+50 mg/kg DDMP	123.20±6.78	190.00±7.04	34.84±5.98

Values are represented in mean values±SEM, n=6

+ *p*<0.05 when compared with controls

PC – *Plukenetia conophora* DDMP - 4H-Pyran-4-One 2,3-Dihydro-3,5-Dihydroxy-6-Methyl

### Effects of oral administration of *Plukenetia conophora* or 4H-Pyran- 4-One 2, 3-Dihydro-3,5-Dihydroxy-6-Methyl on relative reproductive organ weight

Cadmium chloride administration significantly reduced (*p*<0.05) the weight of testes, seminal vesicle, and epididymis respectively, as compared with controls. On the other hand, oral administration of *Plukenetia conophora* or 4H-Pyran-4-One 2,3-Dihydro-3,5-Dihydroxy-6-Methyl significantly improved (*p*<0.05) relative reproductive organ weight when compared with the group given cadmium chloride only (Table 5).

**Table 5:** Effects of oral administration of Plukenetia conophora seedsand4H-Pyran-4-One 2,3-Dihydro-3,5-Dihydroxy-6-Methyl on the relative organ weight of male Wistar rats treated with a single IP dose of 2mg/kg of cadmium chloride.

Group	Testis	Seminal vesicle	Epididymis
Control	0.47±0.09	0.24±0.04	0.26±0.04
Cadmium chloride	0.19±0.03^+^	0.10±0.02	0.13±0.01^+^
Cd+Vit E	0.16±0.04	0.16±0.04	0.14±0.02
Cd+100 mg/kg PC	0.45±0.26	0.15±0.05	0.15±0.01
Cd+200mg/kg PC	0.22±0.02	0.26±0.06	0.10±0.02
Cd+25 mg/kg DDMP	0.20±0.03	0.22±0.04	0.15±0.04
Cd+50 mg/kg DDMP	0.35±0.09^*^	0.23±0.05	0.20±0.05

Values are represented in mean values±SEM, n=6

+ *P<0.05 *when compared with controls

* *p*<0.05 when compared with the group given cadmium chloride alone

PC – *Plukenetia conophora* DDMP - 4H-Pyran-4-One 2,3-Dihydro-3,5-Dihydroxy-6-Methyl

### Effects of oral administration of *Plukenetia conophora* or 4H-Pyran-4-One 2,3-Dihydro-3,5-Dihydroxy-6-Methyl on epididymal semen analysis

Significant decreases (*p*<0.05) in sperm count, motility, and viability were observed in cadmium chloride treated groups as compared with controls. The group pre-treated with cadmium chloride and administered oral *Plukenetia conophora* or 4H-Pyran-4-One 2,3-Dihydro-3, 5-Dihydroxy-6-Methyl showed significant increases (*p*<0.05) in sperm count, motility, and viability when compared with the group given cadmium chloride only (Table 6).

**Table 6:** Effects of oral administration of Plukenetia conophora and4H-Pyran-4-One 2,3-Dihydro-3,5-Dihydroxy-6-Methylon the epididymal semen of male Wistar rats treated with a single IP dose of 2 mg/kg of cadmium chloride.

Group	Count (106cell/ml)	Motility (%)	Viability (%)	Morphology (%)
Control	91.6±4.31	80.0±4.47	93.60±1.57	7.6±1.12
Cadmium chloride	24.2±9.70+	14.4±3.08+	8.60±1.02+	10.8±1.53
Cd+Vit E	45.6±4.31^*^	41.60±13.60^*^	23.60±2.93^*^	6.2±1.36^*^
Cd+100 mg/kg PC	44.0±4.06^*^	30.0±4.47^*^	20.0±1.70^*^	5.8±1.36^*^
Cd+200mg/kg PC	61.0±7.57^*^	34.0±7.48^*^	26.0±1.87^*^	7.8±1.20
Cd+25 mg/kg DDMP	67.0±5.04^*^	32.6±3.60^*^	26.6±2.06^*^	5.6±0.36^*^
Cd+50 mg/kg DDMP	85.0±5.90^**^	68.4±9.80^**^	84.6±5.44^**^	6.8±1.10

Values are represented in mean values±SEM, n=6

+ *p*<0.05 when compared with controls,

* *p*<0.05 when compared with the group given cadmium chloride alone

** *p*<0.01 when compared with the group given cadmium chloride alone

PC – *Plukenetia conophora*, D DMP - 4H-Pyran-4-One 2,3-Dihydro-3,5-Dihydroxy-6-Methyl

### Effects of oral administration of *Plukenetia conophora* or 4H-Pyran-4- One 2,3-Dihydro-3,5-Dihydroxy-6-Methyl on malondialdehyde and nitric oxide levels

Cadmium chloride treated rats showed a significant increase (*p*<0.05) in testicular and epididymal malondialdehyde and nitric oxide levels as compared to controls (Table 7). The group pre-treated with cadmium chloride and administered oral *Plukenetia conophora* or 4H-Pyran-4-One 2,3-Dihydro-3,5-Dihydroxy-6-Methyl showed significant decreases (*p*<0.05) in the testicular and epididymal malondialdehyde and nitric oxide levels as compared with the group given cadmium chloride only (Table 7).

**Table 7:** Effects of oral administration of Plukenetia conophora and 4H-Pyran-4-One 2,3-Dihydro-3,5-Dihydroxy-6-Methylon malondialdehyde and nitric oxide levels of male Wistar rats treated with a single IP dose of 2 mg/kg of cadmium chloride.

Group	Testis		Epididymis	
MDA	NO	MDA	NO
Control	4.36±0.98	2.50±0.33	4.91±1.38	3.13±0.16
Cadmium chloride	29.37±3.58^+^	4.04±0.62^+^	18.91±2.52^+^	3.92±0.02
Cd+Vit E	9.24±0.70^**^	2.79±0.00^*^	6.74±0.22^**^	3.00±0.17^*^
Cd+100 mg/kg PC	6.41±0.88^**^	2.42±0.07^*^	6.06±0.68^**^	3.07±0.56
Cd+200mg/kg PC	5.34±1.32^**^	2.43±0.09^*^	4.51±0.15^**^	2.66±0.22^*^
Cd+25 mg/kg DDMP	10.93±1.82^**^	2.07±0.07^*^	9.51±1.63^**^	3.20±0.28
Cd+50 mg/kg DDMP	4.63±1.12^**^	2.83±0.24^*^	2.47±0.16^**^	3.11±0.00

Values are represented in mean values±SEM, n=6

+ *P<0.05* when compared with controls

* *p*<0.05 when compared with the group given cadmium chloride alone

** *p*<0.01 when compared with the group given cadmium chloride alone

MDA - Malondialdehyde NO - Nitric oxide PC – *Plukenetia conophora* DDMP - 4H-Pyran-4-One 2,3-Dihydro-3,5-Dihydroxy-6-Methyl

### Effects of oral administration of *Plukenetia conophora* or 4H-Pyran-4- One 2,3-Dihydro-3,5-Dihydroxy-6-Methyl on endogenous antioxidant enzymes

Oral administration of *Plukenetia conophora* or 4H-Pyran-4-One 2,3-Dihydro-3,5-Dihydroxy-6-Methyl led to significant increases (*p*<0.05) in the levels of endogenous antioxidant enzymes superoxide dismutase, catalase, glutathione peroxidase, and glutathione-S-transferase in tissue homogenates as compared with the group treated with cadmium chloride only (Tables 8 and 9). The group treated with cadmium chloride had a significant decrease (*p*<0.05) in endogenous antioxidant enzyme levels when compared with controls (Tables 8 and 9).

**Table 8:** Effects of oral administration of Plukenetia conophora and4H-Pyran-4-One 2,3-Dihydro-3,5-Dihydroxy-6-Methylon the testicular antioxidant status of male Wistar rats treated with a single IP dose of 2 mg/kg of cadmium chloride.

Group	SOD (U/ml)	CAT (nmol/min/ml)	GPX (nmol/min/ml)	GST (nmol/min/ml)
Control	60.46±11.0	2.40±0.59	1.03±0.07	0.43±0.19
Cadmium chloride	9.68±4.10^+^	0.89±0.13^+^	0.78±0.01	0.07±0.02^+^
Cd+Vit E	68.00±5.20^**^	1.41±0.14	1.21±0.24	0.26±0.02^*^
Cd+100 mg/kg PC	61.85±12.80^**^	1.91±0.40	1.56±0.28^*^	0.36±0.13^*^
Cd+200mg/kg PC	66.62±4.60^**^	2.31±0.48^*^	1.69±0.26^*^	0.25±0.07^*^
Cd+25 mg/kg DDMP	57.93±9.65^**^	1.94±0.40^*^	1.50±0.11^*^	0.16±0.02
Cd+50 mg/kg DDMP	66.62±4.60^**^	2.65±0.40^*^	1.94±0.26^*^	0.32±0.08^*^

Values are represented in mean values±SEM, n=6

+ when compared with controls,

* **p*<0.05 *when compared with the group given cadmium chloride alone

** **p*<0.01 *when compared with the group given cadmium chloride alone

Superoxide dismutase (SOD) Catalase (CAT) Glutathione peroxidase (GP_X_), Glutathione-S-Transferase (GST) PC – *Plukenetia conophora* DDMP - 4H-Pyran-4-One 2,3-Dihydro-3,5-Dihydroxy-6-Methyl

**Table 9:** Effects of oral administration of Plukenetia conophora and4H-Pyran-4-One 2,3-Dihydro-3,5-Dihydroxy-6-Methyl on the epididymal antioxidant status of male Wistar rats treated with a single IP dose of 2 mg/kg of cadmium chloride.

Group	SOD (U/ml)	CAT (nmol/min/ml)	GPX (nmol/min/ml)	GST (nmol/min/ml)
Control	43.31±4.99	2.02±0.09	2.46±0.49	0.35±0.17
Cadmium chloride	13.89±2.39^+^	0.74±0.13^+^	0.96±0.06^+^	0.08±0.03^+^
Cd+Vit E	30.09±2.71^*^	1.48±0.33^*^	1.07±0.07	0.19±0.02
Cd+100 mg/kg PC	30.88±5.19^*^	1.63±0.18^*^	1.31±0.21^*^	0.21±0.13^*^
Cd+200mg/kg PC	30.88±3.17^*^	2.07±0.06^*^	1.00±0.13^*^	0.18±0.02
Cd+25 mg/kg DDMP	36.29±3.44^*^	1.97±0.15^*^	1.62±0.24^*^	0.27±0.04^*^
Cd+50 mg/kg DDMP	35.13±3.94^*^	2.16±0.08^*^	1.49±0.31^*^	0.24±0.06^*^

Values are represented in mean values±SEM, n=6

+ **p*<0.05* when compared with controls,

* **p*<0.05* when compared with the group given cadmium chloride alone

Superoxide dismutase (SOD) Catalase (CAT) Glutathione peroxidase (GPX) Glutathione-S-Transferase (GST) PC – *Plukenetia conophora* DDMP - 4H-Pyran-4-One 2,3-Dihydro-3,5-Dihydroxy-6-Methyl

### Effects of oral administration of *Plukenetia conophora* or 4H-Pyran-4-One 2,3-Dihydro-3,5-Dihydroxy-6-Methyl on hormone levels

**Figure 4:**
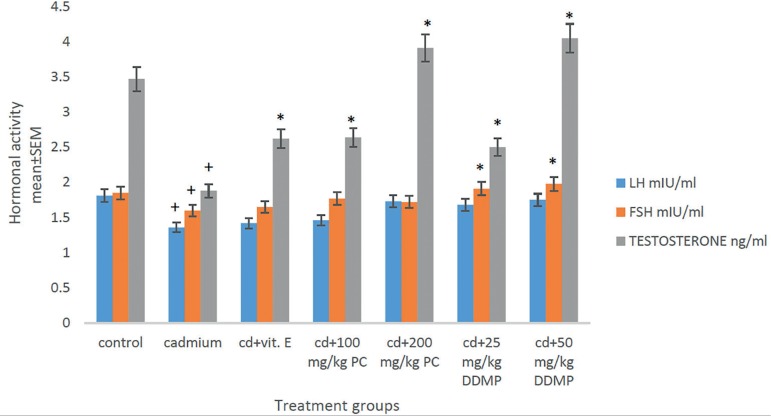
Effects of oral administration of PC and DDMP on hormone levels of male Wistar rats treated with a single IP dose 2 mg/kg of cadmium chloride. **+** *p*<0.05when compared with controls, * *p*<0.05 when compared with the group given cadmium chloride alone PC - *Plukenetia conophora* DDMP - 4H-Pyran-4-One 2,3-Dihydro-3,5-Dihydroxy-6-Methyl

There was a significant increase (*p*<0.05) in testosterone levels in the group administered oral *Plukenetia conophora* or 4H-Pyran-4-One 2,3-Dihydro-3,5-Dihydroxy-6-Methyl as compared with the group given cadmium chloride only (Figure 4). The group treated with cadmium chloride showed a significant decrease in testosterone levels as compared with controls (Figure 4).

### Effects of oral administration of *Plukenetia conophora* and 4H-Pyran-4-One 2,3-Dihydro-3,5-Dihydroxy-6-Methyl on proton pump (ATPase) activity

**Figure 5:**
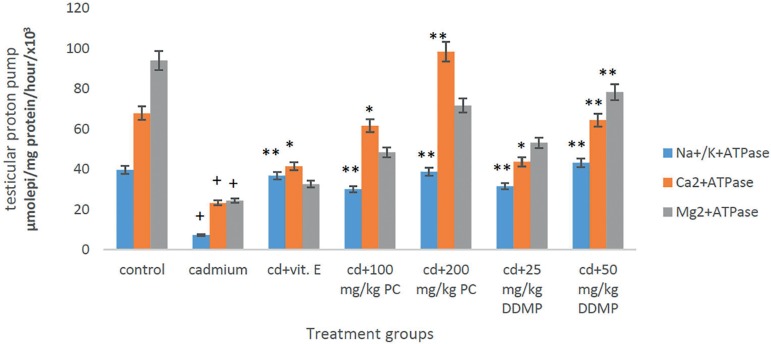
Effects of oral administration of PC and DDMP on the testicular proton pump (ATPase) activity of male Wistar rats treated with a single IP dose of 2 mg/kg of cadmium chloride. **+** when compared with controls *p<0.05 when compared with the group given cadmium chloride alone, ** *p*<0.01 when compared with the group given cadmium chloride alone PC - *Plukenetia conophora* DDMP - 4H-Pyran-4-One 2,3-Dihydro-3,5-Dihydroxy-6-Methyl

**Figure 6:**
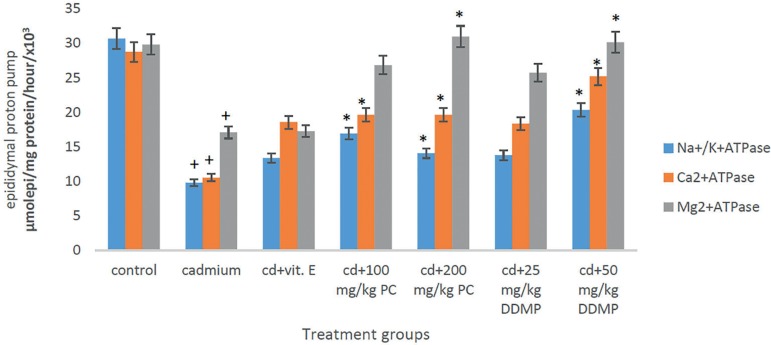
Effects of oral administration of PC and DDMP on the epididymal proton pump (ATPase) activity of male Wistar rats treated with a single IP dose of 2 mg/kg of cadmium chloride. **+** *p*<0.05 when compared with control * *p*<0.05 when compared with the group given cadmium chloride alone, PC - *Plukenetia conophora* DDMP - 4H-Pyran-4-One 2,3-Dihydro-3,5-Dihydroxy-6-Methyl

In this study, significant decreases (*p*<0.05) were observed in the levels of activity of the membrane bound enzymes (ATPase) in the testes and epididymis of rats treated with cadmium chloride when compared with controls (Figures 5 and 6). Significant increases in the level of activity of these enzymes was observed in the group treated with oral *Plukenetia conophora* or 4H-Pyran-4-One 2,3-Dihydro-3,5-Dihydroxy-6-Methyl when compared to the group given cadmium chloride only (Figures 5 and 6).

## DISCUSSION

Cadmium, a heavy metal, is a major environmental toxicant found in both developed and developing countries (de Souza Predes *et al*., 2010). Cadmium is discharged in the environment due to the use of petroleum products, combustion of fossil fuels (petroleum and coal), and municipal refuse, all of which contribute to airborne cadmium pollution (Hutton & Symon, 1986; Ragan & Mast, 1990; De Rosa *et al.,* 2003). Cadmium has long been established as a reproductive toxicant because of its damaging effects on the male reproductive organs (Gagnon, 1988; Toppari *et al.,* 1996). It destroys the testes, accumulates in semen, and causes endocrine disruption; its adverse effects on hormone concentration, testicular enzyme activities, sperm parameters, and male infertility in general have been documented (Benoff *et al*., 2000; Kini *et al*., 2011).

The result of this study showed that the group treated with cadmium chloride showed significant reductions (*p*<0.05) in body weight gain as compared with controls, a finding previously reported in the literature (de Souza Predes *et al.,* 2010). The group pre-treated with cadmium chloride and administered oral *Plukenetia conophora* or 4H-Pyran-4-One 2,3-Dihydro-3,5-Dihydroxy-6-Methyl showed improved body weight gain as compared with the group treated with cadmium alone (Table 4). This may be explained by the presence of carbohydrates and flavonoids in the extract.

Cadmium chloride administration significantly reduced (*p*<0.05) the weight of testes, seminal vesicle, and epididymis. This finding may also be related to decreased feeding, as even mild food restriction results in lower serum levels of testosterone and luteinizing hormone and lower weight of androgen-dependent organs (Trentacoste *et al.,* 2001). These observations were supported by our results. The weight of the testes is largely dependent on the mass of differentiated spermatogenic cells; reduction in testis weight may be due to decreased number of germ cells, which can cause inhibition of spermatogenesis and steroidogenic enzyme activity (Takahashi & Oishi, 2001). The observed loss of weight of accessory sex organs may be due to reduced bioavailability of sex hormones (Schrade, 2003). On the other hand, oral administration of *Plukenetia Conophora* or 4H-Pyran-4-One 2,3-Dihydro-3,5-Dihydroxy-6-Methyl significantly improved (*p*<0.05) reproductive organ weight (Table 5). These effects might be due to the presence of carbohydrates and flavonoids in the extract.

A significant reduction (*p*<0.05) in sperm count, motility, and viability were observed in cadmium chloride treated groups as compared with controls. The group treated with oral administration of *Plukenetia Conophora* or 4H-Pyran-4-One 2,3-Dihydro-3,5-Dihydroxy-6-Methyl had significant increases (*p*<0.05) in sperm count, motility, and viability when compared with the group treated with cadmium chloride (Table 6). These effects may be explained by the androgen biosynthesis capacity of the extract originated from the high flavonoid content needed in normal testicular function.

The sperm plasma membrane has a high content of polyunsaturated fatty acids susceptible to lipid peroxidation by oxidative stress (Agarwal & Prabakaran, 2005). Estimating end products of lipid peroxidation such as malondialdehyde serves as indication of the extent of oxidative damage to cellular structures (Sharma & Agarwal, 1996). The rats treated with cadmium chloride showed a significant increase (*p*<0.05) in testicular and epididymal MDA and NO levels as compared with controls (Table 7). This increase might be attributed to the concomitant increase in generation of free radicals (ROS) by cadmium chloride such as peroxide radicals in the testes and epididymis, thereby causing lipid peroxidation and reduction in antioxidant levels of tissue homogenates (Table 8). This may be the initial event in the production of testicular and epididymal damage by cadmium that might be linked to the deleterious effects this heavy metal has on reproductive organ weight, sperm parameters (sperm count, sperm motility, and viability), antioxidant status, and hormonal disturbances (see Tables 5, 6, 7 and 8 and Figure 4, respectively). This may be caused by the exhaustion of antioxidant enzymes and the consequent inability to catalyse the overproduction of hydrogen peroxide caused by cadmium chloride toxicity. Oral administration of vitamin E, *Plukenetia Conophora* or 4H-Pyran-4-One 2,3-Dihydro-3,5-Dihydroxy-6-Methyl significantly reduced (*p*<0.05) testicular and epididymal MDA and NO levels (Table 7). The effect might be attributed to the high content of flavonoid fraction present in the extract.

The testes have an elaborate antioxidant defence mechanism that involves superoxide dismutase, catalase, glutathione peroxidase, and glutathione-S-transferase, which react with hydrogen peroxide to preventing intracellular damage caused by oxidative stress. Several studies have reported decreased activities of endogenous antioxidant enzymes in cases of exposure to heavy metals and cadmium chloride in particular (Kini *et al.,* 2009; 2011). Oral administration of *Plukenetia conophora* and 4H-Pyran-4-One 2,3-Dihydro-3,5-Dihydroxy-6-Methyl led to significant increases (*p*<0.05) in endogenous antioxidant enzymes in tissue homogenates (Tables 8 and 9). These effects may be due to presence of high content of flavonoid in *Plukenetia conophora* extract.

Testosterone is the main steroid sex hormone in male Wistar rats; it is secreted by Leydig cells in the testes under the control of complex neuroendocrine interactions (Popa *et al.,* 2008). High level of testosterone in the testes is critically required for normal spermatogenesis, development, and maintenance of sperm morphology and normal physiology of the seminiferous tubules (Weinbauer *et al.,* 2010). The significant increase (*p*<0.05) in the level of testosterone in the group administered oral *Plukenetia Conophora* or 4H-Pyran-4-One 2,3-Dihydro-3,5-Dihydroxy-6-Methyl may stem from the testicular androgen biosynthesis capacity of the extract due to its high flavonoid content (Figure 4).

Results from several studies have implicated decreased proton pump (ATPases) activity in male reproductive dysfunction (Vujisić *et al.,* 2004). Oxidative stress has been suggested as a contributory factor to impaired ATPase activity. Proton pumps maintain trans-membrane gradients for the ions and produce a convenient driving force for the secondary transport of metabolic substrates such as amino acids and glucose. ATPases are responsible for proper cellular function and for preserving the ionic gradient across the cell membrane, membrane potential, and osmotic equilibrium, thus allowing the transportation of Na^+^, K^+^, Ca^2+^, Mg^2+^ ions across the membrane at the expense of ATP hydrolysis in the testes and epididymis (Vujisić *et al.,* 2004). In this study, significant decreases (*p*<0.05) in the level of activity of the membrane bound enzymes (ATPase) in the testes and epididymis of rats treated with cadmium chloride was observed (Figures 5 and 6). These effects might be attributed to altered membrane fluidity, enhanced lipid peroxidation, and declining antioxidant defence status in the male reproductive organ. ATPase has been shown to be very susceptible to free radicals and membrane lipid peroxidation (Misra *et al.,* 1998). Lipid peroxidation has been shown to alter Na^+^/K^+^-ATPase, calcium ATPase and magnesium ATPase functions by modification at specific active sites in a selective manner (Qayyum *et al.,* 2001). Depletion of glutathione and other protective antioxidants by ROS may greatly contribute to increase the levels of reactive species, which may also account for impaired activity of Na^+^/K^+^-ATPase (D’Ambrosio *et al.,* 2001). Significant increases in the level of activity of these enzymes was observed in the group treated with oral *Plukenetia Conophora* or 4H-Pyran-4-One 2,3-Dihydro-3,5-Dihydroxy-6-Methyl as compared to the group given cadmium chloride (Figures 5 and 6). The modulation of the activity of the three membrane-bound ATPases by the extract seen in this study suggests vital roles for the plant in the maintenance of sperm variables in the testes.

## CONCLUSION

*Plukenetia conophora* alleviated male reproductive toxicity induced by cadmium chloride in Wistar rats. The observed effects might be attributed to its high flavonoid content (4H-Pyran-4-One 2,3-Dihydro-3,5-Dihydroxy-6-Methyl).
